# Understanding Cervical Cancer through Proteomics

**DOI:** 10.3390/cells10081854

**Published:** 2021-07-22

**Authors:** Fátima Martínez-Rodríguez, Jared E. Limones-González, Brenda Mendoza-Almanza, Edgar L. Esparza-Ibarra, Perla I. Gallegos-Flores, Jorge L. Ayala-Luján, Susana Godina-González, Eva Salinas, Gretel Mendoza-Almanza

**Affiliations:** 1 Microbiology Department, Basic Science Center, Autonomous University of Aguascalientes, Aguascalientes 20100, Mexico; al260704@edu.uaa.mx; 2 Master in Biomedical Sciences, Autonomous University of Zacatecas, Zacatecas 98160, Mexico; 33143655@uaz.edu.mx; 3 Academic Unit of Biological Sciences, Autonomous University of Zacatecas, Zacatecas 98068, Mexico; brenda.mendoza@uaz.edu.mx (B.M.-A.); lesparza@uaz.edu.mx (E.L.E.-I.); ivonne_gf@uaz.edu.mx (P.I.G.-F.); 4 Academic Unit of Chemical Sciences, Autonomous University of Zacatecas, Zacatecas 98160, Mexico; jayala69@uaz.edu.mx (J.L.A.-L.); sgodina@uaz.edu.mx (S.G.-G.); 5 National Council of Science and Technology, Autonomous University of Zacatecas, Zacatecas 98000, Mexico

**Keywords:** proteomic, cervical cancer, biomarkers, gene differential expression

## Abstract

Cancer is one of the leading public health issues worldwide, and the number of cancer patients increases every day. Particularly, cervical cancer (CC) is still the second leading cause of cancer death in women from developing countries. Thus, it is essential to deepen our knowledge about the molecular pathogenesis of CC and propose new therapeutic targets and new methods to diagnose this disease in its early stages. Differential expression analysis using high-throughput techniques applied to biological samples allows determining the physiological state of normal cells and the changes produced by cancer development. The cluster of differential molecular profiles in the genome, the transcriptome, or the proteome is analyzed in the disease, and it is called the molecular signature of cancer. Proteomic analysis of biological samples of patients with different grades of cervical intraepithelial neoplasia (CIN) and CC has served to elucidate the pathways involved in the development and progression of cancer and identify cervical proteins associated with CC. However, several cervical carcinogenesis mechanisms are still unclear. Detecting pathologies in their earliest stages can significantly improve a patient’s survival rate, prognosis, and recurrence. The present review is an update on the proteomic study of CC.

## 1. Introduction

Cervical cancer (CC) is the second most common form of cancer in women living in developing countries [1]. Diagnostic tests such as Pap smears and viral DNA analysis, and the development of vaccines against different genotypes of human papillomavirus (HPV), have significantly contributed to reducing the incidence of CC. However, it still causes a high number of deaths among vulnerable populations of women [2].

In general, CC is defined as a multi-stage process involving the uncontrolled proliferation of malignantly transformed cells. Phenotypically, it starts with early tissue alterations known as hyperplasia; it then progresses to dysplasia, cancer in situ, and finally to invasive cancer that can spread to tissues near and far through the lymphatic and blood systems and metastasize [3,4,5].

Each genotypic and phenotypic change gives way to alterations in the tumor microenvironment and tumor progression [6]. It has been observed that all types of cancer share genetic alterations, especially in the signaling pathways that control cell progression, apoptosis, and cell growth [7,8]. The most significant alterations include: (1) alterations in the mechanisms responsible for maintaining and repairing DNA through mutations that can be hereditary or sporadic and can occur in all somatic and germ cells [9]; (2) transformation of proto-oncogenes into oncogenes through mutations that change the structure, location, expression, or function of genes [10]; (3) mutations that suppress the activity of tumor suppressor genes [11].

The proteins encoded by tumor suppressor genes are classified according to the influence they exert as follows: (a) proteins inside the cell that inhibit cellular replication at a particular stage of the cell growth and division cycle [12]; (b) proteins that act as receptors and bind to hormones or chemical signals that tell the cell not to divide [13]; (c) proteins that stop cell division when the DNA has been damaged [14], some examples of proteins encoded by tumor suppressor genes involved in cancer development are p53, Rb, BRCA1, BRCA2, PTEN, SMAD2, and SMAD4 [12,13,14,15,16]; (d) alterations in the regular expression of proteins from signaling pathways involved in cell development, such as RTK/RAS/MAP-Kinase, PI3K/Akt, Myc, Notch, TGFβ, ß catenin, Wnt, and Hippo, among others [17,18,19].

Another aspect of the carcinogenesis process has been elucidated in recent years. Cancer stem cells (CSC) express proteins associated with self-renewal, high plasticity, migration capacity, and telomerase expression [6,20]. It has been proven that the transition between the mesenchymal and epithelial states, considered the primary step in the metastasis process, is an acquired capacity of CSC [21]. The main proteins involved are Snail, Twist, Slug, and BMI-1, among others [6,20,21,22,23]. This finding has research and clinical importance since CSC are considered crucial in cancer growth, metastasis, invasion, resistance to chemotherapy, recurrence, and poor prognosis [6,20].

There is a broad spectrum of molecular alterations generated by cancer. However, not all are necessary for determining cancer cell phenotypes or the development, progression, and degree of cancer malignancy [24].

High-throughput techniques applied to biomedical data and the analysis of various biological samples such as tissue [25], plasma, serum [26], cervicovaginal fluid [27], or cervical swabs [28], have allowed us to determine the physiological state of cells, together with their microenvironment, in normal conditions, and also the way they change during the development of pathological conditions. These techniques also allow us to discriminate between significant and non-significant cell alterations during the development of a disease, identifying the molecular signature of the disease in the genome, transcriptome, and proteome.

This review aims to summarize the technological developments and scientific contributions in proteomics that have made it possible to deepen our understanding of the pathogenesis of CC.

## 2. Proteomics

Proteomics is the systematic and quantitative identification of the complete set of proteins (proteome) present in a biological system (a cell, a tissue, an organ, a biological fluid, or an organism) at a specific point in time [29,30,31]. The concept of the proteome encompasses all proteins that can be studied after being generated by alternative splicing and post-translational modifications [32]. Thus, proteomics studies decipher not only changes in protein expression levels, but also post-translational modifications that are essential to the regulation of protein functions. The main objective of proteomics is to develop a comprehensive understanding of biological systems by studying all the proteins that make up, for example, a cell [33]. The study of proteomics includes proteome characterization, protein expression abundance, protein production rates, protein degradation, stability, abundance, structure and function, post-translational modifications, protein–protein interactions, migration through subcellular compartments, and the relationship between proteins and different metabolic pathways [34,35].

Conventional protein analysis techniques using 1D SDS-PAGE gels, Western Blot, or ELISA do not allow for the massive analysis of proteins or determine protein expression levels precisely. However, they still generate valuable information and are helpful in the validation of data generated by Mass Spectrometry (MS) or two-dimensional gels electrophoresis (2-DE) [36,37].

The first proteomic studies were carried out in 1975 by three independent researchers, O′ Farrell, Klose, and Scheele. They pioneered the mapping of *Escherichia coli*, mouse, and guinea pig proteins, respectively, on two-dimensional gels. They managed to separate a large number of proteins, but they were not able to identify them [38]. From that starting point, proteomics has evolved mainly due to advances in the precision, sensitivity, and speed of analytical instruments and advances in bioinformatics that allow us to collect, store, process, and visualize the vast amount of generated data in proteomic studies [39,40,41].

**Figure 1:**
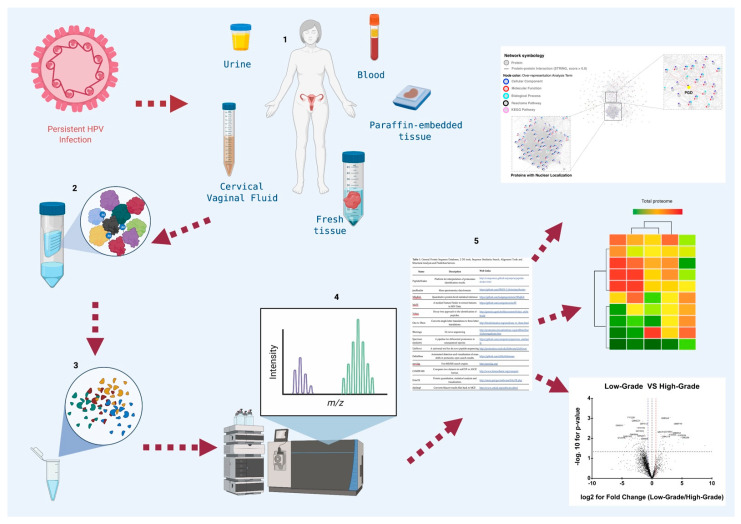
General diagram of the workflow in proteomic studies in cervical cancer. Proteomic studies in cervical cancer are designed to search for biomarkers that could be used in the diagnosis, prognosis, and identification of new therapeutic targets, and they are carried out Figure 1. (1) Collection of biological samples from patients and controls such as: urine, vaginal cervical fluid, blood, and tumor tissue. If the samples start from cells, it is essential to perform cell lysis or mechanical destruction (freeze thaw, French press, sonication macerated with liquid N_2_, lysis with detergents). (2) Purification of total proteins by centrifugation. The storage of the sample until its use at temperatures of −20 °C or −70 °C with or without protease inhibitor according to the chosen step of proteomic analysis to avoid any interference with the selected method. (3) Proteomic strategy selection, based on advanced techniques: protein microarray, mass spectrometry, Edman sequencing, 2D gel, 2D-DIGE. Quantitative techniques: ICAT, SILAC, iTRAQ. High throughput techniques: X-ray crystallography, NMR spectroscopy. (4) Match in databases and validation of candidates by ELISA, Western Blot or Immunohistochemistry. (5) Graphics construction. Partially created with BioRender.com.

A typical proteomic experiment involves several steps (Figure 1) [42,43,44,45], depending on the purification method used according to the protein’s location, its abundance, size, known or expected load, the intended application of the purified protein, the laboratory budget, and infrastructure. Depending on the proteomic technique selected, the results obtained by each laboratory may vary.

Globally, proteomics can characterize the function and location of proteins and is broadly divided into three categories: (1) Functional Proteomics, which refers to various proteomic approaches that allow the study and characterization of protein signaling, the mechanisms of protein-related disease, and protein–drug interactions [46]. (2) Structural Proteomics is the study of the subcellular location of proteins and protein–protein interactions through the purification of organelles or complexes and the subsequent identification of their components by MS [47]. (3) Expression Proteomics is the quantitative study of the expression of proteins among samples that differ in some variable, comparing the total proteome or subproteomes between different samples [48]. Thus, the obtained information can identify new proteins involved in signal transduction or the development of a disease [49].

Comparative proteomics is a branch of quantitative proteomics that analyzes changes in the proteome in response to different stimuli produced by the environment or a disease [50,51]. It is used in the analysis and search of cancer biomarkers and relies on reproducible techniques of sample selection, purification, and storage [45,51]. The main techniques used for the large-scale analysis of comparative data are 2-DE and MS [28,51]. MS acquires the spectral data of peptides (from digested proteins), and bioinformatics tools are then necessary to identify the peptides and corresponding consensus proteins, such as MASCOT, Protein Pilot, Skyline, and DAVID Bioinformatics Resources, among others. They are then compared with human protein databases such as Swiss-Prot or UniProt and classified according to their function and subcellular location [52].

### 2.1. Two-Dimensional Gels Electrophoresis

Smithies and Poulik proposed using 2-DE gels in 1956, which are based on the combination of two electrophoretic processes to obtain a higher resolution in the separation of protein mixtures [53]. 2-DE gels allow the separation of 4000 to 5000 proteins in a single experiment by separating proteins according to their isoelectric point and then separating these proteins according to their molecular mass on SDS-PAGE gel [54]. These gels also allow the characterization of post-translational modifications, mutant proteins, and differential protein expression in pathological states [55].

The main advantage of this technique relies on facilitating the visualization of the protein profile, which facilitates making a direct comparison with other protein maps [56]. However, it also has several disadvantages that make it of little value in the search of possible cancer biomarkers: it is challenging to automate; very large or hydrophobic proteins do not enter the gel during the first-dimension electrophoresis, while very acidic or fundamental proteins are not well resolved (for example, the proteins of cell membranes, which represent 40% of all cell proteins). It also struggles to detect rare proteins, some of which are very important (regulatory proteins, proteins involved in signal transduction, receptors) [57,58,59].

Several modifications have been made to the original 2-DE gels technique, including the pre-staining of samples with fluorophores, which increases the technique’s sensitivity and is known as two-dimensional differential in-gel electrophoresis (2D-DIGE) [60]. This variation makes it possible to perform a comparative proteomics analysis, in a quantitative point-by-point manner, of peptides or proteins absent or present in greater or lesser amounts in one sample compared to another [60,61]. It is a more sensitive, precise, and reproducible technique. Fluorescent dyes such as Cy2, Cy3, and Cy5 are used to tag proteins in the samples before the electrophoresis run, and it is possible to run more than one sample with different dyes and to obtain results with less variation [60,61].

### 2.2. Mass Spectrometry

Proteomics is currently a crucial part of biological and biomedical research. The most-used tool for identifying and quantifying the set of proteins contained in a proteome is liquid chromatography, followed by mass spectrometry (LC-MS) [62,63,64], a technique so sensitive that it can detect the least abundant proteins guaranteeing a great depth of analysis.

Mass spectrometry is based on measuring the mass/charge ratio (*m*/*z*), which helps determine the molecular weight of proteins [65]. The technique comprises three critical processes:

(1) Ionization of molecules. Integrating the ionization of molecules to MS drastically increased the sensitivity and depth of proteome analysis [65]. The ionization methods most commonly used in comparative proteomics and the ones with the highest sensitivity, precision, and reproducibility are matrix-assisted laser desorption ionization (MALDI) [66], surface-enhanced laser desorption/ionization (SELDI) [67], and electrospray ionization (ESI) [68]. (2) The separation of ions according to the ratio between their *m*/*z* values. (3) Measurement of the ions separated according to the *m*/*z* ratio. Currently, the most widely used MS kits for proteomic analysis are LC-MS/MS [69] and MALDI time-of-flight (TOF)/TOF [70].

Ionization by MALDI does not require much work before analysis. The samples are mixed with a chemical matrix followed by the application of a high-energy laser that generates ions. These types of experiments are conveniently performed on simple protein samples. MALDI is a modern technique for the identification and detection of microorganisms [69,71].

SELDI ionization is an easy-to-use variation of MALDI. It binds proteins to a surface with a substrate, thus eliminating interferences. It is widely used to detect biomarkers in various diseases, such as cancer, due to its high throughput and good sensitivity to low-molecular-weight peptides [67,72,73]. SELDI detects small (~500 Da) or truncated peptides in minimal sample volumes (2 µL). It is not recommended for samples that contain high molecular weight proteins, as it struggles to detect them [73].

ESI has been a crucial piece in the revolutionary success of MS. This technique allows biomolecules to reach a convenient liquid phase for the first step of liquid chromatography and then move to a gaseous phase, which is the phase required for MS analysis [67,74]. The father of ESI, John B. Fenn, won the Nobel Prize in Chemistry in 2002, shared with Koichi Tanaka, for discovering MALDI [75].

TOF is another mass spectrometer that increases the sensitivity and efficiency of biomarker screenings. It relies on detecting specific peptides that result from a fragmentation process in scanning-type experiments called “precursor ion scanning”. In these experiments, the triple quadrupole keeps constant the last mass filter and scans ranges of parental *m*/*z* values associated with the desired fragment [76,77,78,79].

The variation in the efficiency of ionization methods increases the complexity of quantitative proteomic analysis by MS [80]. However, emerging marking techniques such as chemical labeling and labeling with stable isotopes contribute to make MS an accurate quantitative technique [81]. The main quantitative proteomic methods involve the following techniques: isotope-coded affinity tag (ICAT) labeling [82], stable isotope labeling with amino acids in cell culture (SILAC) [83,84], and isobaric tag for relative and absolute quantitation (iTRAQ) [85].

ICAT is a quantitative method based on isotopic labeling in vitro. ICAT binds by affinity cysteine residues of normal or denatured proteins [86]. It can be used to designate appropriate biomarkers for cancer diagnosis [45]. ICAT reagents react with thiol groups of cysteine residues. When coupled with MS, this technique is helpful for the quantification of proteins with thiol groups, but it is useless for analyzing proteins in the proteome that do not have cysteine residues in their composition, approximately 10% of proteome proteins [87]. SILAC is an MS-based quantitative proteomics approach used for internal labeling of the cell proteome or of proteins secreted into the cell culture supernatant [83]. It is a type of labeling proteomics that can aid in the analysis of gene expression, cell signaling, and post-translational modifications [88]. It is also beneficial for the analysis of protein secretion pathways and the proteins thus secreted [89]. iTRAQ allows for multiplex labeling when making a relative or absolute MS-based quantification of proteins. This method analyzes N-terminal and amino groups labeled in protein chains that are fractionated through LC and analyzed by MS [90]. The importance of ITRAQ analysis is that it allows the analysis of the mechanisms of disease development by simultaneously identifying and quantifying proteins. The data resulting from these techniques are analyzed by bioinformatics to identify proteins, measure their abundance, and assess their relationship with signaling pathways using specific databases. The development of new algorithms for massive data analysis has increased the specificity and precision of identifying and quantifying proteins (Table 1).

## 3. Proteomics and Cervical Cancer

Despite the significant advances that have been made in the diagnosis of CC, such as Pap cytology (Pap), colposcopy, visual inspection with acetic acid (VIA), and histopathological examination, the development of molecular techniques such as polymerase chain reaction (PCR), hybridization, and sequencing, and the development of treatments such as radiotherapy, chemotherapy, surgery, immunotherapy, and targeted therapy, it is still the cause of millions of deaths each year worldwide, especially in developing countries [91,92,93]. Through the International Agency for Research on Cancer, the World Health Organization reported a cancer incidence rate in women of 604,127 new cases throughout the world in 2020 and a mortality rate of 341,831 women around the world [93]. CC continues to be the type of cancer with the second-highest incidence and mortality in women worldwide. Africa, Latin America, and Asia are the three continents with the highest incidence and mortality rates due to CC [93].

Proteomic analysis could be of great help in the fight against CC as it allows the monitoring of changes in protein levels, which can lead to the discovery of new biomarkers of CC, increasing the chances of an early diagnosis with a good prognosis, and even of the development of new effective therapies based on these biomarkers [94,95].

The main obstacles in the proteomic study of patient samples with any disease are the patient physiology (co-morbidities, co-infections, pregnancies), sample preparation, and protein separation and identification methods [96,97]. Several studies have shown that different separation and identification methods are associated with significant differences in the proteins identified in the same type of sample (same type of biological sample and disease stage) and disease [48,98,99].

One of the main problems in the study of biomarkers is the limited number of available samples, which increases the chance of false-positive candidates [99,100]. In CC, the type of biological samples that are most useful for finding biomarkers are tumor tissue, cervical, vaginal fluid, blood, and even saliva. Significant advances have been made in developing and implementing proteomic techniques to obtain valuable information on CC (Table 2).

### 3.1. Biological Samples in Proteomic Studies of CC

#### 3.1.1. Cervical Cancer Cell Line

The proteome of CC has been studied in cell lines associated with CC, such as HeLa, SiHa, and CaSki mainly. It has also been studied in samples of patients with different stages of CC using 2-DE gels and MS. Some proteomic studies carried out on CC cell lines are summarized below. In 2011, Higareda-Almaraz et al. [103] studied a proteomic pattern in CC cell lines to identify common cellular events associated with CC. The proteomic techniques used were 2-DE gels and MALDI-TOF MS. They identified a core of 66 proteins associated with the development of CC. They reported that the main functions of these proteins are related to cell migration, adhesion, epithelial-mesenchymal transition, metastasis, evasion of apoptosis, and energy metabolism enzymes [103].

Another strategy for studying CC based on proteomics is the study of cell membranes as possible therapeutic targets. In 2018, Pappa et al. [105] published a work on the isolation and enrichment of membrane proteins of three different cell lines, HeLa, SiHa, and C33A CC. They performed a proteomic characterization of these cell lines by LC-MS/MS and a bioinformatics analysis using Proteome Discoverer 1.4, SEQUEST, and UniProt. The Mann–Whitney statistical analysis used to compare the results with the non-cancer cell line HCK1T allowed them to identify 263 unique transmembrane proteins in C33, 262 unique transmembrane proteins in HeLa, and 152 unique transmembrane proteins in SiHa. Among the identified transmembrane proteins, TMX2, FAM120A, CLPTM1, CKAP5, and NCSTN were the most prominent proteins differentially expressed in CC cell lines [105].

In 2020, Xia et al. [106] published a study using iTRAQ-based quantitative proteomic analysis to analyze the effect of metformin on invasion and migration of the CC cell lines HeLa and SiHa. The mechanism by which metformin inhibits the proliferation and invasion of CC cells was analyzed. After treatment with metformin, the authors found 53 differentially expressed proteins, 20 overexpressed proteins, and 33 under-expressed proteins. Proteomic analysis, complemented with tumor xenograft modeling, showed that the expression of nine proteins was decreased in cells treated with metformin, namely TGFβ-1, CCPG1, LGMN, SLC38A2, TRIM26, MTR, ATP6AP1, CIRBP, and PTP4A1, while the expression of CYR61 and IGFBP7 was increased compared to control cells. The authors concluded that metformin was capable of inhibiting the proliferation and invasion of CC cells in this proteomic assay [106].

#### 3.1.2. Cervical-Vaginal Fluid

Cervical-vaginal fluid (CVF) is a non-constant biological fluid influenced by hormones, menstrual cycles, age, microbiota, immunological state, and sexual activity. CVF plays a crucial role in protecting the vagina from pathogenic microorganisms. The microbiota present in this fluid make it an essential source of information about the woman’s immune status and the precancerous or cancerous cervical state [4]. CVF is an ideal biological fluid to study and identify biomarkers for the early diagnosis of CC [15]. An essential fact in the role of CVF as a source of CC biomarkers is that most proteins are present in the cytoplasm or the extracellular region (21 and 20%, respectively), and the precancerous or cancerous tissue is in direct contact with the CVF [17].

The main techniques used to study the proteome in CVF include the pre-fractionation of samples by high-performance liquid chromatography (HPLC), reverse-phase liquid chromatography (RPLC), and protein identification MALDI-TOF/TOF mass spectrometry. Some examples of proteomic studies in CVF are summarized below.

In 2007, Shaw et al. [128] used a “bottom-up” proteomics approach to characterize the protein repertoire of human CVF by 2-DE followed by one-dimensional-SDS-PAGE. MALDI-TOF/TOF identified the spots, and the bioinformatics analysis was performed using the Ingenuity software. The authors identified a total of 685 proteins, some of which were confirmed by ELISA. They reported defense-related proteins such as haptoglobin, defensins, lactoferrin, azurocidin, and dermcidin. They also identified serine and cysteine proteases such as Kallikrain-related peptidases 6, 7, 10, 11, 12, and 13 [128].

In 2009, Zegels et al. [129] used (RP)-LC MALD I-TOF/TOF to identify 339 proteins in human CVF, including antimicrobial peptides such as human beta-defensin-2 and cathelicidin. The first time the technique was applied, 151 proteins were identified, while 136 had previously been reported, including extracellular proteins with immunological functions [129].

Another way to use CVF to analyze the proteome of patients with different grades of CC is the study of exosomes, which are small vesicles that contain proteins, lipids, and nucleic acids in space and time determined by what is a representative sample of the state of the cell from which they come [3,4]. It is well known that the mRNA and miRNA contained in exosomes constitute a specific molecular signature that can characterize a patient′s pathological state. Exosomes also carry double-stranded DNA, and, in the case of cancer patients, the exosomes released by malignant cells contain precious information, including proteins. So far, there are no reports on the proteome of exosomes from the CVF of patients with different stages of CC.

In 2021, Boylan et al. [28] reported a proteomic study in samples from patients with advanced CC. The samples were collected by Pap smear, cervical swab, or from ovarian tumor tissue. The proteins in each sample were digested with trypsin and analyzed using 2D-LC MS/MS. The data were analyzed using UnitPro, Scaffolf v.4.8.2, while PANTHER was used to identify peptides and proteins and to analyze their location and molecular function. The three types of biological samples were found to contain 2293 proteins in common, 490 differentially expressed proteins in tumor tissue, 64 differentially expressed proteins in the Pap test fluid, and 320 differentially expressed proteins in a cervical swab. The tumor tissue and the cervical swab had 1423 proteins in common, the tumor tissue and pap samples shared 186 proteins, and 158 proteins were shared by swab and pap samples, concluding that Pap test fixatives and cervical swabs are a rich source of tumor-specific biomarkers for ovarian cancer [28].

In 2020, Ma et al. [134] published a proteomic study of cervical adenocarcinoma in situ in which the proteome of normal cervical samples was compared with endocervical adenocarcinoma samples using iTRAQ marking followed by LC-MS-TOF. Cervical adenocarcinoma and endocervical adenocarcinoma are the two main types of CC, with the highest prevalence in young populations. The analysis identified 711 proteins, of which 237 were differentially expressed in endocervical adenocarcinoma, while 256 proteins were differentially expressed between adenocarcinoma in situ and control samples. Furthermore, 242 proteins were differentially expressed between adenocarcinoma in situ and endocervical adenocarcinoma. Gene ontology (GO) analysis performed on 1056 differentially expressed proteins showed that the highest percentages corresponded to proteins related to metabolic processes, cellular processes, biological regulation, response to stimuli, and biological regulation processes. The authors concluded that APOA1 might be a candidate marker for cervical adenocarcinoma and a study target to determine the functional mechanisms of this disease [134].

#### 3.1.3. Cervical Cancer Tissue

Tissue from patients with CC used in biomedical research is obtained after the patients sign letters of consent and the Bioethics Committee approves the research protocol of the hospital that collaborates with the research. Fresh samples are much easier to process than formalin-fixed, paraffin-embedded (FFPE) tissue samples that were obtained from CC patients 5, 10, or 12 years ago, depending on the protection policies of each hospital. Using PET samples has the drawback that the proteins can be degraded in the treatments (deparaffinization and cell lysis) before analyzing the proteins. The advantage is that it is possible to obtain a much more robust number of samples from different stages of CC.

Some examples of proteomic studies carried out in CC tissue are summarized below.

In 2005, Bae et al. [115] compared the profiles of proteins from CC biopsies with proteins from healthy tissue. 2-DE and MALDI-TOF performed the analysis, and the resulting data were analyzed using Mascot, Swiss-Prot, and the NCBI-nr database. The authors identified 35 proteins in CC tissue, 17 of which were found up-regulated and 18 down-regulated [115].

Gu et al. in 2007 [116] reported the results of a proteomic analysis of CC cells from tissue samples with a high degree of cervical dysplasia, which the authors compared to normal cervical tissue samples. Laser Capture Microdissection, combined with LC-MS, was used to identify more than 200 proteins in cancer cells. Overall, a significant up-regulation of nuclear and mitochondrial proteins was found in samples of high-grade squamous cervical intraepithelial lesions compared to normal cervical epithelial cells [116].

In 2009, Zhu et al. [117] reported the differential expression of proteins Tyk2, S100A9, and zinc-finger protein 217 in squamous CC, compared to unaffected adjacent cervical tissue, according to 2D-DIGE and MALDI-TOF MS. The data were analyzed using the MASCOT program and the NCBI-nr database. They concluded that these proteins could have a potential application for diagnosis and therapy [117].

In 2014, Wang et al. [25] studied three proteins related to metastatic processes in CC, FABP5, HspB1, and MnSOD. They analyzed tissue samples with and without pelvic lymph node metastasis as detected by 2D-DIGE and MALDI-TOF/TOF MS. Data analysis was performed using MASCOT and the BioTools software [25].

In 2015, Zao et al. [118] published their research on the differentially expressed proteins between a normal cervix, cervical intraepithelial neoplasia (CIN), and cervical squamous cell carcinoma (CSCC). The analysis was performed in normal cervical tissue, CIN, and CSCC samples using 2D-DIGE and the DeCyder software. MALDI-TOF/TOF MS was then used to identify the differentially expressed proteins. The results were validated by Western Blot (WB) and immunohistochemistry (IHC). The results showed that the S100A9 protein was the most significantly up-regulated protein among the three samples. IHC showed that protein S100A9 was mainly expressed in the cytoplasm and that its positive expression rate was 20.0% in the normal cervix, 70.0% in CIN, and 100.0% in CSCC, with significant differences between them (*p* = 0.006). Other proteins, such as eEF1A1, which was the most significantly down-regulated protein, were found mainly expressed in the cell plasma; the positive expression rate of eEF1A1 was 70.0% in the normal cervix, 73.3% in CIN, and 60.0% in CSCC, without significant differences between them (*p* = 0.758). PKM2 was mainly expressed in cell nuclei; its positive expression rate was 100.0% in the normal cervix, 93.3% in CIN, and 75.0% in CSCC, with differences close to statistical significance (*p* = 0.059) between them. According to the authors, these three proteins could be candidate markers for the early diagnosis of CC and new targets for therapy. These proteins could also serve as the basis of new studies on the molecular mechanisms that participate in CIN, CSCC [118].

In 2016, Serafín-Higareda [122], performed a comparative analysis, using 2D-DIGE and MALDI-TOF, of six cases of cervical HPV-16 and four surgical specimens without lesions related to CC and without HPV infection. To identify differential protein profiles, they used Decyder Software. They identified three proteins that were overexpressed in CC: Mimecan, Actin, and Lumican. They also found that keratin, type II cytoskeletal 5, Peroxiredoxin-1, and 14-3-3 protein sigma reduced their protein expression level in CC compared to normal cervix cells [122].

In 2017, Qing et al. [130] published a study on the proteome associated with HPV-16 infection and the development of CC. They used iTRAQ-based proteomic analysis to reveal the regulatory network of proteins expressed in cervical carcinoma associated with HPV infection to find possible biomarkers for the diagnosis of HPV-associated cancer. Bioinformatic analysis was performed using the MetaCore^TM^ software. The authors concluded that, when overexpressed, proteins ASAH1, PCBP2, DDX5, hnRNPA1, MCM4, MCM5, CYC, ENO1, and TYPH could be potential biomarkers for CC and high-risk HPV infections [130].

In 2018, Güzel et al. [119] carried out a study in CC tissue and healthy tissue using shotgun proteomics techniques combined with nano LC-MS/MS to analyze tissue lysate digest samples. An average of 1700 proteins were identified in each sample. According to the differential expression between CC and healthy tissue, several proteins were found to be significantly up-regulated, found by analysis with Scaffold and Mascot software. MCM4 was found in the early and late stages of CC but was not detectable in healthy tissue, while ENDOU, MT-ND4, and RDH12 were only detected in healthy tissue [119].

Hwang et al. [135] reported 30 proteins that were differentially expressed in CC samples according to 2-DE-MALDI-TOF-MS. The most crucial protein identified by the study was HSP60, as validated by WB, and was proposed by the authors as a marker of CC prognosis [135].

Proteomics studies of CC have also been used to study and analyze proteins involved in resistance to chemo or radiotherapy. In this sense, in 2020, Chel Hun Choi et al. [136] published a work on predictive models of radioresistance based on a protein panel constructed from 181 samples of patients with advanced CC. After a reverse-phase protein assay (a method to detect and quantify low-abundance proteins with high sensitivity and precision) in tumor samples, and validation by WB, the authors found that proteins BCL2, HER2, CD133, CAIX, and ERCC1 are predictors of survival in advanced CC patients, which may be helpful in identifying the response to chemoradiation [136].

#### 3.1.4. Blood

All components of blood are widely used for disease diagnosis and prognosis. Many biomarkers have been found in plasma and serum in many parts of the world. Biomedical researchers have made significant efforts so that the biomarkers found in various blood components can be used as a much less invasive diagnostic method called liquid biopsy. However, confirming a pathology and its biomarkers in the blood is highly complicated due to the low concentration range of biomarkers in several blood components.

Cancer antigens are usually circulating in the blood at a concentration of ng/mL. The characterization of these proteins using powerful tools as 2-DE gels combined with MS identification can be performed by comparing the profiles of serum samples from CC patients with healthy controls.

In 2014, Boichenko et al. [26] published a study on 84 serum samples from healthy controls, 16 samples from patients with CIN, 23 from patients with early CC, and 20 from patients with advanced CC. The authors analyzed the proteome of these samples by iTRAQ labeling followed by LC-MS/MS. LC QTOF MS/MSCLS conducted the confirmation of the findings. The analysis of the results was performed in both cases using MassHunter Qualitative Analysis, Phenix, X! Tandem, Scaffold, and UniPro. In the first analysis, the authors found the following proteins: HPT, A1AT, TRFE, FETUA, A1AG1, AACT, KNG1, and VTDB. They also analyzed serum samples from patients with late-stage ovarian cancer to test the specificity of the selected biomarkers for CC. After the analysis, the authors concluded that A1AG1, A1AT, and HPT are not specific to CC [26].

In 2015, Guo et al. [137] analyzed early-stage cervical carcinoma samples using 2D-DIGE gels followed by MALDI-TOF MS. After analyzing the obtained data using the DeCyder 2D software, ten proteins were identified that could be used as possible CC biomarkers, including proteins related to lipid metabolism, apolipoprotein A-IV (APOA4), apolipoprotein A1 (APOA1), and apolipoprotein E (APOE); metabolic enzymes such as ceruloplasmin (CP), endoglycosidase F2, mannan-binding lectin-associated serine protease 2 (MASP2), and CLU glycoprotein; and proteins related to immune functions. The networks in which the analyzed proteins participate are related to lipid metabolism and molecular transport [137].

## 4. Other Omics Studies in Cervical Cancer

Undoubtedly, the participation of other omics techniques has been crucial in advancing the knowledge of CC.

### 4.1. Genomics

Genomics has helped to understand the genetic changes that facilitate cancer development. Studies that can be performed range from whole-genome studies, specific gene profiling, comparisons between the tumor cell genome and cells, detection of rare somatic variants, tumor sub-clones, and circulating DNA fragments [138].

Genomic DNA is the key to gene expression since its replication is finely programmed in space and time where a series of factors intervene, such as replication loci or the opening of chromatin to carry out replication and the transcription [139].

Throughout the history of DNA studies, excellent qualitative and quantitative characterization methods have been developed, such as quantification of nucleotide analog incorporation and DNA copy number analysis, and in more recent years, massive sequencing genome has made it possible to evolve in the type of analysis and the understanding of DNA replication mechanisms.

In advances in CC knowledge, genomics has served to clarify mutations/polymorphisms that generate susceptibility to the development of the disease, and the effect of DNA methylation in CpG-rich promoter regions, such as hypomethylation, general loss of methylation during CC carcinogenesis, and gene silencing by hypermethylation of tumor suppressor genes [140].

The Cancer Genome Atlas Research Network has been dedicated to identifying new genomic and molecular characteristics of CC for their molecular classification and the creation of targeted therapies [141]. From genomic analysis, they discovered that about 5% of primary CCs were not caused by persistent HPV infection and might be triggered by genetic alteration or other factors. They found that TP53, PTEN, CTNNB1, ARID5BA, and ARID1A are cancer-driver genes. Other important conclusions are that the CDO1, PCDHB2, and MYOD1 genes have a different response to radiotherapy depending on HPV presence or absence, and in this sense, that RP11-299 L17.3, SLC14A2, FGF18, and OASL have a different response to cisplatin. Furthermore, the PIK3CA, PTEN, TP53, STK11, KRAS, SHKBP1, ERBB3, HLA-A, CASP8, and TGFBR2 genes are significantly mutated in CC [141].

On the other hand, DNA methylation plays an essential role in genome stability and gene expression. It is heritable and does not change the DNA sequence. Hypermethylation of tumor suppressor genes has been widely characterized as one of the first steps in human carcinogenesis [140].

In a normal cell, tumor suppressor genes are hypomethylated or unmethylated, in contrast to the tumor cells in which the hypermethylation of CpG islands in promoter regions of tumor suppressor genes prevents their transcription. It has been demonstrated that methylation increases with the progression of CIN to CC in tissues. In in vitro models, several genes have been recognized as methylation targets, such as miR124-2, CADM1, MAL, and PAX1, and have been corroborated in patient samples [142].

From genome sequencing with platforms such as Illumina Infinium MethylationEPIC BeadChip, it has been possible to identify the functional effects of the methylation of long non-coding RNAs (lncRNA) in cervical carcinogenesis. Wang et al. reported a methylation map using RNA sequencing (RNA-seq). They identified 3962 hypermethylated CpG sites cancer, 363 up-regulated and 664 down-regulated lncRNAs [143]. Of all the lncRNAs identified, according to the analysis of the Kaplan–Meier survival curves, they showed that only the lncRNA SOX21-AS1 had clinical prognostic value in CC [143].

On the other hand, MeiGong et al. [144] conducted a study on polymorphisms, demonstrating the relationship of polymorphisms of methylenetetrahydrofolate reductase (MTHFR) and CC analysis in 372 samples of women. They concluded that the MTHFR A1298C polymorphism was significantly higher in the cancer group than in the control group, and its presence was associated with a risk of developing CC, but not with CIN [144].

### 4.2. Metagenomic

Microbiomes have taken an essential significance by associating the microorganisms present with the development of some diseases, including cancer.

There is a solid and clear association between the uterine and vaginal microbiome present and the development of CC. The predominance in the vaginal microbiota of *Lactobacillus* species is associated with maintaining a healthy reproductive status. At the same time, the results found in later stages of CIN and CC vary, but among other genera, the most reported are *Serratia* and *Gardnerella* [145].

Usyk et al. [146] conducted a study on the role of the cervicovaginal microbiome in the natural history of incident infection by high-risk HPV in 273 women from Costa Rica between 18 and 25 years of age. The work aimed to analyze the elimination capacity, persistence, and progression of the infection to CIN 2 and 3. The authors characterized the microbiome by amplifying and sequencing the bacterial 16S rRNA V4 gene region and the ITS1 fungal region using an Illumina MiSeq platform. They observed that the abundance of *Lactobacillus iners* was associated with eliminating incident high-risk HPV infections. At the same time, *Gardnerella* was a dominant biomarker for the progression of HPV to CIN 2 and CIN 3 [146].

Kwon et al. [147] identified cervical microbiome alterations in CC in Korean women using metagenome sequencing. While in CIN 2 and 3, samples were characterized by *Lactobacillus*, *Staphylococcus*, and *Candidatus Endolissoclinum*, the control group was enriched with *Pseudoalteromonas* and *Psychrobacter*. Furthermore, in CC, the genera *Alkaliphilus*, *Pseudothermotoga*, and *Wolbachia* were the most enriched [147].

The same authors reported the pyrosequencing of the 16S rRNA gene in previous work. They demonstrated that cervical microbial patterns rich in *Atopobium vagiane*, *Lactobacillus iners*, and *Gardnerella vaginalis* and not rich in *Lactobacillus crispatus* had a high risk of a CIN progression [148].

Mitra et al. [149] carried out a study related to the changes in intestinal microbial diversity with the toxicity caused by chemoradiation therapy for CC from stool sampling obtained from thirty-five patients subjected to chemoradiation and analyzed by 16S RNA sequencing. The results obtained indicate that the gut microbiome continuously decreases during chemoradiation therapy, with the most significant decrease in the fifth week. There is a strong correlation between good gastrointestinal functioning and a greater diversity of the intestinal microbiome, while highly toxic patients demonstrated different compositional changes during chemoradiation therapy, in addition to compositional differences in *Clostridia* species [149].

### 4.3. Transcriptomic

The transcriptome is the complete set of transcripts in a cell at a specific time and under a physiological condition. By understanding the transcriptome, the functional elements of the genome can be interpreted. It allows us to know the repertoire of genes expressed in the host–pathogen interaction, splicing-alternative patterns, and quantification of the changes in expression levels that can be carried out during a given moment or specific condition [150].

Li et al. [151] analyzed the aberrant expression patterns of CC using RNA-Seq data from the Cancer Genome Atlas. They identified the genetic signature of the histone family by integrating genetic profiles, molecular signatures, and functional and pathway information with enrichment network analysis of gene sets and protein–protein interaction. From this analysis, it was revealed that DNA repair systems were significantly correlated with survival rate. In addition, they provided evidence that the sets of SLe-associated genes HIST1H2BD, HIST1H2BJ, HIST1H2BH, HIST1H2AM, and HIST1H4K can be used as prognostic factors for predicting survival in patients with CC [151].

Xu et al. [152] analyzed the transcriptomes of normal cervical tissue and CC tissue samples positive for HPV and identified 614 differentially expressed transcripts between the different stages of cervical cancer. They observed that LY6K, FAM83A, CELSR3, ASF1B, IQGAP3, SEMA3F, CLDN10, MSX1, CXCL5, ASRGL1, ELAVL2, GRB7, KHSRP, NOVA1, and RNiH2 could be new biomarkers of progression in CC. They analyzed that the presence of HPV16 or 18 could alter the expression of CDKN2A, ELAVL2, GRB7, HSPB1, KHSRP, NOVA1, PTBP1, and RNASEH2A in human vaginal keratinocytes and foreskins [152].

Brant et al. [153] reported one of the few studies on the expression of the papillomavirus genome and the frequency of alternately spliced E6/E7 mRNA in invasive CC. They comprehensively characterized the expression of HPV by RNA-Seq analysis in 22 biopsies of invasive CC with HPV16 or HPV18, characterizing the presence of integrated/episomal viral DNA, the integration sites in the human genome, and the proportion of products of alternative splicing of genes E6 and E7. Their results demonstrated the presence of viral DNA integrated into the human genome in most tumors [153].

Hua et al. [154] identified several HPV16 E7 regulated genes with putative roles in tumorigenesis. They performed digital RNA sequencing, and a total of 195 differentially expressed genes were identified between NHEK cells transfected with HPV16 E7 and control cells. They found that the differentially expressed genes, IFI6, SLC39A9, and ZNF185, showed a strong correlation with tumor progression, while AKAP12 and DUSP5 have a crucial role in carcinogenesis and poor prognosis, as has been previously established for other types of cancer [154].

### 4.4. Metabolomics

Metabolomics involves the detailed investigation of metabolites and small molecules closely related to the disease. In CINs and CC, it has been used extensively to study cancer metabolism and identify biomarkers indicative of disease, states, and underlying etiology [155].

Khan et al. [156] identified unique metabolic signatures for CIN and CC by using global and specific metabolic profiles from examination of 69 normal plasma samples, 55 CIN 1, 42 CIN 2, CIN 3, and 60 CC samples using Ultra-Performance Liquid Chromatography, Quadrupole Time-of-Flight Mass Spectrometry (UPLC-QTOF-MS) together with multivariate statistical analysis. They identified 28 metabolites that had discriminatory levels between normal, CIN, and CC patients, finding the metabolic pathways of alanine, aspartate, and glutamate significantly altered. Specifically, the metabolites AMP, aspartate, glutamate, hypoxanthine, lactate, proline, and pyroglutamate were significantly high in patients compared to normal controls and were associated with an increased risk of developing CIN 2/3 and CC [156].

Abudula et al. [157] explored the prediction of CC from potential biomarkers based on the metabolic profile of cervical and uterine tissue samples that were positive for HPV16 infection. They used 21 tissues from patients with CSCC, 20 from CIN 2 and 3, and 11 control samples. High-resolution magic angle spinning nuclear magnetic resonance was used to analyze the metabolic profile in the tissues along with conventional tests such as PCR and IHC for validation. A profile of 17 small molecular weight metabolites was identified that showed differential expression in CC or CIN 2 or 3 positive for HPV16, compared to the control group. They observed a significant increase in GSK3β and GAD1 at levels of transcription and protein. Additionally, decreased transcription and protein levels of PKM2 and CPT1A were reported. Based on the results, the authors conclude that HPV infection and cervical carcinogenesis cause metabolic modifications that could be associated with aberrant regulation of enzymes related to metabolic pathways [157].

Paraskevaidi et al. [158] evaluated a novel method for CC screening. Using an automated metabolomic robotic platform and the principle of laser-assisted rapid evaporative ionization mass spectrometry (LA-REIMS), a population of 130 women was analyzed. LA-REIMS achieved 94% sensitivity and 83% specificity in the detection of high-risk HPV positive women. Therefore, the authors conclude that the use of platforms such as LA-REIMS can further improve the accuracy and efficiency of the current national detection program [158].

## 5. Concluding Remarks

Advances in analytical techniques and bioinformatics provide a broad spectrum of alternatives to carry out proteomic studies. The proteomic analysis of CC can now be performed on any biological sample (tissue, blood, urine, saliva, vaginal fluid). Each type of sample represents a potential source of diagnostic and prognostic biomarkers and potential therapeutic targets.

A proteomic analysis may reveal the presence and absence of a protein or a treatment effect on tumor shrinkage; it can predict a patient′s prognosis or elucidate the transduction networks in which a given protein participates and how it does so. The results obtained by MS and their analysis using different databases made possible to identify the signaling pathways and specific genes that participate in the development and progression of cancer in metastatic processes, angiogenesis, and recurrence.

The recurring problem with the proteomic studies focused on finding potential disease biomarkers is the high variability of the results reported by each laboratory. There is variability in the concentrations reported and even in the type of biomarker identified, even when different research groups work with the same biological samples. Different techniques have been developed to reduce or eliminate this variability in the results of proteomic studies, such as the combination of technologies such as MS and isotopic or chemical labeling, or, more recently, label-free protein quantification using LC−MS/MS.

It would be beneficial to integrate all the information generated so far by proteomic studies worldwide and pay special attention to the validation of the proteins that different researchers have identified as potential biomarkers for the diagnosis and prognosis of possible new therapeutic targets.

## Figures and Tables

**Table 1:** General Protein Sequence Databases, 2-DE tools, Sequence Similarity Search, Alignment Tools and Structural Analysis and Prediction Servers.

Name	Description	Web Links
PeptideShaker	Platform for interpretation of proteomics identification results	http://compomics.github.io/projects/peptide-shaker.html
jmzReader	Mass spectrometry data formats	https://github.com/PRIDE-Utilities/jmzReader
MSqRob	Quantitative protein-level statistical inference	https://github.com/ludgergoeminne/MSqRob
MoFF	A modest Feature Finder to extract features in MS1 Data.	https://github.com/compomics/moFF
Nokoi	Decoy-free approach to the identification of peptides.	http://genesis.ugent.be/files/costore/Nokoi_utilities.zip
One to Three	Converts single-letter translations to three-letter translations.	http://bioinformatics.org/sms2/one_to_three.html
Sherenga	De novo sequencing	http://proteomics.broadinstitute.org/millhtml/batchsherengaframe.htm
Spectrum similarity	A pipeline for differential proteomics in unsequenced species.	https://github.com/compomics/spectrum_similarity
UniNovo	A universal tool for de novo peptide sequencing.	http://proteomics.ucsd.edu/Software/UniNovo/
DeltaMass	Automated detection and visualization of mass shifts in proteomic open search results.	https://github.com/chhh/deltamass
greylag	Free MS/MS search engine.	http://greylag.org/
COMSPARI	Compares two datasets in netCDF or ASCII format.	http://www.biomechanic.org/comspari
DAnTE	Protein quantitation, statistical analysis and visualization.	http://omics.pnl.gov/software/DAnTE.php
dat2mgf	Converts Mascot results files back to MGF.	http://www.ce4csb.org/software.shtml
DataAnalysis2TPP	Converts MGF from Bruker DataAnalysis to TPP-friendly format for use with XPRESS and ASAPRatio	http://www.ms-utils.org/DataAnalysis2TPP.html
MS-Spectre	Quantitiave analysis of multiple LC-MS(/MS) analyses in mzXML.	http://sourceforge.net/projects/ms-spectre
msaccess	Creates a pseudo-2D-gel representation.	http://proteowizard.sourceforge.net/tools/msaccess.html
mspire_mspire-sequest	MS data processing in Ruby, including mzML reader/writer/converter, ‘‘in-silico’’ digestion, isotopic pattern calculation etc.	http://mspire.rubyforge.org
Multi-Q	Tool for multiplexed iTRAQ-based quantitation.	http://ms.iis.sinica.edu.tw/Multi-Q/
mzBruker	Converts analysis.baf files from Bruker into mzXML files. This software requires the CDAL library from Bruker	http://tools.proteomecenter.org/wiki/index.php?title=Software:mzBruker
MzJava	Library for the analysis of mass spectrometry data from large scale proteomic and glycomics experiments	http://mzjava.expasy.org/
PROTICdb	Proteomic database to store, track, query and compare proteome data.	http://pappso.inra.fr/bioinfo/proticdb/
ProtMAX	Fast and robust software tool for analyzing large shotgun proteomics mass spectrometry data sets.	http://www.univie.ac.at/mosys/software.html
MassWolf	Converts MassLynx format to mzXML	http://tools.proteomecenter.org/MassWolf.php
mres2x	A tool to process MASCOT results.	https://sourceforge.net/projects/protms/files/mres2x/
mzXML2	Converts mzXML and mzML files to SEQUEST dta, MASCOT mgf, and Micromass pkl files.	http://tools.proteomecenter.org/wiki/index.php?title=Software:MzXML2Search
nontarget	R function for compound, adducts and ion series detection using isotopic distributions.	http://cran.r-project.org/web/packages/nontarget/index.html
PAPPSO	Southwest Paris proteomic analysis platform.	http://pappso.inra.fr/bioinfo/
PEAKS De Novo	Integrated ‘‘de novo’’ peptide sequencing, PTM finder, and homology search (demo available).	http://www.bioinfor.com/peaks/features/denovo.html
QuPE	Web application to support the analysis and integration of even complex mass spectrometry-based proteomics experiments	https://qupe.cebitec.uni-bielefeld.de
Skyline	Builds SRM/MRM methods and analyzes resulting data.	https://brendanx-uw1.gs.washington.edu/labkey/project/home/software/Skyline/begin.view
unfinnigan	Reading Thermo raw files without MsFileReader.	https://code.google.com/p/unfinnigan/
ThermoRawFileParser	Open-source, crossplatform tool that converts Thermo RAW files into open file formats such as MGF and to the HUPO-PSI standard file format mzML	https://github.com/compomics/ThermoRawFileParser
BatchServer	Web application for batch effect evaluation, visualization and correction.	https://lifeinfo.shinyapps.io/batchserver/
psims	Prototype work for a unified API for writing PSIMS standardized XML documents, currently just mzML and MzIdentML.	https://github.com/mobiusklein/psims
Compomics sigpep	Predicting peptide signatures for targeted proteomics.	https://github.com/compomics/compomics-sigpep
DACSIM	De novo peptide sequencing based on a divide-and-conquer algorithm and peptide tandem spectrum simulation	https://pubs.acs.org/doi/abs/10.1021/ac0491206
Dinosaur	Peptide feature detector for LC-MS data	https://github.com/fickludd/dinosaur/
Isoform Resolver	A peptide-centric algorithm for protein inference.	https://www.ncbi.nlm.nih.gov/pmc/articles/PMC3167374/
Jtraml	Java implementation of the PSI-MS Transitions Markup Language (TraML) specification.	https://github.com/compomics/jtraml
Protein Disorder	List is a list of Protein Disorder Predictors	http://www.disprot.org/predictors.php
Protein	Monthly review written by the Swiss-Prot team of the Swiss Institute of Bioinformatics. Spotlight articles describe a specific protein or family of proteins on an informal tone.	https://web.expasy.org/spotlight/
Proteins API	Provide sequence feature annotations from UniProtKB, variation data from UniProtKB and mapped from LSS.	http://www.ebi.ac.uk/proteins/api
Proteinspector	Analysis of mass spectrometry proteomics quality control metrics.	https://bitbucket.org/proteinspector/qc_analysis/
raw2mzDB	An extension of the ProteoWizard framework enabling the support of the mzDB format.	https://github.com/mzdb/pwiz-mzdb
SeqMS	De novo sequencing by tandem mass spectrometry	https://www.ncbi.nlm.nih.gov/pubmed/10870956/
msInspect	A software platform for rapidly creating computational tools for mass spectrometry-based proteomics.	https://github.com/dhmay/msInspect
Peptizer	Automating manual validation of MS/MS search results.	https://github.com/compomics/peptizer
NIBR 2D-	World-wide gel-based proteomics database.	http://www.expasy.org/world-2dpage/
OMSSA Parser	Java based parser for Open Mass Spectrometry Search Algorithm; omx files.	http://compomics.github.io/projects/omssa-parser.html

**Table 2:** Discovery of deregulated proteins in the different stages of CC analyzing different biological samples by proteomic tools.

Proteins	Sample	Assay/Technique	OE/SE	Conclusion	Reference
CERVICAL CANCER CELL LINE					
DSG2	HeLa, C33A	WB, qPCR	SE	Possible therapeutic target	[101]
MAGE-A3	HeLA, SiHa, C33A, End1/E6E7	WB	OE	Possible therapeutic target, prognostic	[102]
14- 3-3ζ	HeLa, CaLo, SiHa, CasKi, ViBo, C-33A	2D, MALDI-TOF-MS	OE	14- 3-3ζ belong to “central core of CC”	[103]
CD71+, HPV-E6	C33A, C4-1, CaSki	FC, Microarrays WB	OE	Possible therapeutic target	[104]
TMX2, FAM120A, CLPTM1, CKAP5, NCSTN	HeLa, SiHa, C33A	LC-MS/MS	OE	Possible therapeutic target	[105]
TGFβ-1, CCPG1, LGMN, SLC38A2, TRIM26, MTR, ATP6AP1, CIRBP, PTP4A1, CYR61, IGFBP7	HeLa, SiHa	WB iTRAQ-MS	SE:TGFβ1,CCPG1LGMN, SLC38A2, TRIM26, MTR, ATP6AP1, CIRBP, PTP4A1 OE:CYR61, IGFBP7	Possible therapeutic target	[106]
FRESH TISSUE					
SND1	CC and control tissue	qRT-PCR WB	OE	Possible therapeutic target, prognostic	[107]
Occludin	CC and control tissue	IHC	OE	Diagnostic	[108]
HSP70	CC and control tissue	IHC	OE	Possible therapeutic target, diagnostic	[109]
AIF-1, ALP-2, B-FABP, NCK-1, ICA69, PRSS1, CDK4.	CC and control tissue	ESI-MALDI-TOF-MS RT-PCR WB, IHC	OE	Diagnostic	[110]
G6PD	CC and control tissue	iTRAQ NanoLC-MS/MS qRT-PCR. WB Microrray	OE	Possible therapeutic target,	[111]
iRhom1 e iRhom2.	CC and control tissue	IHC, WB	OE	Possible therapeutic target, diagnostic, prognostic	[112]
BIM	CC and control tissue	IHC	OE	Possible therapeutic target, diagnostic, prognostic	[113]
PTK7	CC and control tissue	IHC qRT-PCR.	OE.	Possible therapeutic target, diagnostic	[114]
35 proteins	CC and control tissue	MALDI-TOF-MS	OE: 17 proteins SE: 18 proteins	Diagnostic	[115]
200 proteins	CC and control tissue	LC-MS	OE	Diagnostic, prognostic	[116]
Tyk2, S100A9, ZNF217	CC and control tissue	2D-DIGE MALDI-TOF MS	OE	Possible therapeutic target, diagnostic	[117]
FABP5, HspB1, MnSOD	CC and control tissue	2D-DIGE MALDI-TOF/TOF MS	OE	Diagnostic, prognostic	[25]
S100A9, eEF1A1, PKM2	CC and control tissue	2-D DIGE MALDI-TOF/TOF MS WB, IHC	OE: S100A9, PKM2 SE:eEF1A1	Possible therapeutic target, diagnostic	[118]
MCM4, MT-ND4, RDH12	CC and control tissue	Nano LC- MS/MS	OE	Diagnostic	[119]
FORMALIN-FIXED, PARAFFIN-EMBEDDED (FFPE) TISSUE					
hTERT	FFPE	IHC	OE	Possible therapeutic target, diagnostic	[120]
FasL y TIL	FFPE	IHC	OE: FasL SE: TIL	Possible therapeutic target, diagnostic	[121]
Mimecan, Aortic Smooth Muscle Actin, Lumican, Keratin, Type II Cytoskeleton 5, Peroxyredoxin-1, Sigma 14-3-3	FFPE	IHC, 2-D, MALDI-TOF-MS	OE:Mimecan, Aortic Smooth Muscle Actine, Lumican SE: Keratin, Cytoskeleton 5, Peroxyredoxin-1, Sigma 14-3-3	Possible therapeutic target, diagnostic	[122]
ADAM9	FFPE	IHC	OE	Possible therapeutic target, diagnostic, prognostic	[123]
SEL1, Notch3, SOCS3	FFPE	IHC	OE	Diagnostic, prognostic	[124]
Ebp1	FFPE	IHC	OE	Diagnostic	[125]
CERVICAL VAGINAL FLUID (CVF)					
alpha-actinin-4	CVF	MALDI-TOF-MS, ELISA	OE	Prognostic	[126]
haptoglobin, defensins, lactoferrin, azurocidin dermcidin, KLKs 6, 7, 10, 11, 12, 13	CVF	2D- MALDI-TOF/TOF-MS ELISA	OE	Diagnostic, prognostic	[127]
beta-defensin-2, cathelicidin	CVF	(RP)-LC MALDI-TOF/TOF-MS	Present	Prognostic.	[128]
ASAH1, PCBP2, DDX5, hnRNPA1, MCM4, MCM5, CYC, ENO1, TYPH	CVF	iTRAQ-MS	OE	Diagnostic, prognostic	[129]
SLeA, p53, HPV16 L1	Swab vaginal	ELISA, WB	OE: p53, HPV16 L1 SE: SLeA	Diagnostic, prognostic	[130]
SERUM AND PLASMA					
HPT, A1AT, TRFE, FETUA, A1AG1, AACT, KNG1, VTDB	Serum	iTRAQ labelling. LC-MS/MS. Nanochip LC qTOF MS/MSCLS	OE	Diagnostic	[26]
Gelsoline y Ceruloplasmine	Serum	2D, MS, ELISA, IHC	OE: Gelsoline SE: Ceruloplasmine	Prognostic	[131]
F9, CFI, AFM, HPR, ORM2	Plasma	LC-MS/MS	OE	Diagnostic, prognostic	[132]
URINE					
MMRN1, LRG1, S100A8, SERPINB3, CD44	Urine	LC-MS/MS WB	OE: MMRN1, LRG1 SE: S100A8, SERPINB3, CD44	Diagnostic	[133]

Abbreviations: OE: over expression; SE: sub-expression; WB: Western Blot; qPCR: Quantitative Polymerase Chain Reaction; IHC: immunohistochemistry; FC: flow cytometry.

## Data Availability

Not applicable.
